# Prevalence and risk factors of frailty among home care clients

**DOI:** 10.1186/s12877-017-0660-8

**Published:** 2017-11-17

**Authors:** Minna Miettinen, Miia Tiihonen, Sirpa Hartikainen, Irma Nykänen

**Affiliations:** 1 Kuopio Research Centre of Geriatric Care, School of Pharmacy, Faculty of Health Sciences, University of Eastern Finland, P.O.B 1627, -70211 Kuopio, FI Finland; 2 Institute of Public Health and Clinical Nutrition, School of Medicine, University of Eastern Finland, P.O.B 1627, -70211 Kuopio, FI Finland

**Keywords:** aCGA, Aged, Home care, Malnutrition, Risk of malnutrition

## Abstract

**Background:**

Frailty is a common problem among older people and it is associated with an increased risk of death and long-term institutional care. Early identification of frailty is necessary to prevent a further decline in the health status of home care clients. The aims of the present study were to determine the prevalence of frailty and associated factors among 75-year-old or older home care clients.

**Methods:**

The study participants were 75-year-old or older home care clients living in three cities in Eastern and Central Finland. Home care clients who had completed the abbreviated Comprehensive Geriatric Assessment (aCGA) for frailty (*n* = 257) were included in the present study. Baseline data were obtained on functional status, cognitive status, depressive symptoms, self-rated health, ability to walk 400 m, nutritional status, drug use and comorbidities.

**Results:**

Most of the home care clients (90%) were screened for frailty using the aCGA. Multivariate analysis showed that the risk of malnutrition or malnutrition (OR = 4.27, 95% CI = 1.56, 11.68) and a low level of education (OR = 1.14, 95% CI = 1.07, 1.23) were associated with frailty.

**Conclusion:**

Frailty is a prevalent problem among home care clients. The risk of malnutrition or malnourishment and a lower level of education increase the risk of frailty. Screening for frailty should be done to detect the most vulnerable older people for further intervention to prevent adverse health problems.

**Trial registration:**

ClinicalTrials.gov: NCT02214758.

## Background

The focus of care has shifted to home care instead of residential care, and about 12% of the Finnish population 75 years old or over are home care clients [1]). In Finland, home care services have been provided by municipal social services and healthcare in collaboration with the private sector and non-profit organisations [1].

Home care includes home help, support and nursing with treatments and administering of medication. [1]. Support services include catering, cleaning, laundry, shopping and transport [1]. These services are supplemented by personal home service and are often one of the first services that the client needs to support independent living.

Frailty is an important geriatric syndrome that includes sarcopenia and loss of functional abilities among older people [2–5]. It accelerates ageing, predisposing individuals to adverse health outcomes [2–4]. However, the frailty syndrome is multidimensional and comprises, in addition to physical, also psychological and sociological components [6]. Most studies have been focused on physical weakness and a disease-related view of frailty [2, 3], which does not take into account these psychological or social factors. Separating frailty from normal ageing can be difficult, especially if the method emphasizes physical functions. Frailty has been shown to increase the risk of mortality and institutionalisation [2, 4, 7, 8]. It has been suggested that all persons 70 of age or older should be screened for frailty [9].

Older people prefer living in their own home instead of institutional care [10]. To be able to respond to this is important to prevent further decline in health status, so home care clients should be screened for frailty [2, 4, 11, 12]. The prevalence of frailty has differed in previous studies due to the different definitions used. The abbreviated Comprehensive Geriatric Assessment (aCGA) has been used as a screening tool as it is brief and therefore inexpensive [13, 14]. Most studies have focused on institutionalised or home-dwelling older people. More knowledge and understanding is needed to prevent a decline in clients’ health status. The aims of the present study were to determine the prevalence of frailty and identify the demographical, clinical and functional factors associated with frailty in a population-based sample of home care clients aged 75 or over.

## Methods

### Design and participants

This study is part of the larger Nutrition, Oral Health and Medication (NutOrMed) study. The participants were 75-year-old or older home care clients living in three cities in Eastern and Central Finland Home care clients [15]. The study population in two communities was a randomised sample of home care clients and in one community, a total sample of home care clients. In all, 440 participants were selected and home care nurses asked these clients if they were willing to participate. If the client was cognitively impaired, his or her proxy made the decision on participation. A total of 300 home care clients or their proxies gave written consent for the study. After that, 25 refused to participate, 4 died and 3 moved to another residence, while 11 had missing data. Complete baseline data on aCGA frailty was gathered from 257 participants. A more detailed description of the NutOrMed data is given in a previous article [15].

### Measurements

Nurses, nutritionists and pharmacists conducted interviews in the subjects’ homes. The nurses knew their clients and the nutritionists and pharmacists had previous experience in interviewing and assessing older persons. The interviews concerning sociodemographic factors, activities of daily living and instrumental activities of daily living, cognitive functioning, depressive symptoms and health status were carried out by a home care nurse. If a participant was not able to reply to the interview questions, a caregiver or nurse was interviewed.

Functional ability was assessed with Barthel’s activities of daily living (ADL) index (scale 0–100) [16] and Lawton’s instrumental activities of daily living (IADL) (scale 0–8) [17] and cognitive status was measured with the Mini-Mental State Examination (MMSE) on a scale of 0–30 [18], with higher scores indicating better functioning. The 15-item Geriatric Depression Scale (GDS-15) [19] was used to assess depressive symptoms. Self-rated health was determined using a 5-step scale (very good, good, moderate, poor, very poor), with the two last steps resenting poor health, and self-rated ability to walk 400 m using a 4-step scale (able to walk without difficulties, able to walk with help, unable to walk independently, unable to walk), with the two first steps representing independently [20].

A validated and standardised Mini Nutritional Assessment (MNA) test was used to measure nutritional status, with scores of 24.0–30.0 indicating normal nutritional status and scores <23.5, malnutrition or its risk [21]. The MNA test contains anthropometric measurements, questions on dietary habits, a global assessment and a self-assessment. A pharmacist collected information on drug use; this information was complemented with medication lists, packages and prescriptions. We used a cut-off of 10 drugs/day (excessive polypharmacy) according Jyrkkä et al. [22] because of the high number of drugs used among this population (mean 8.6), thereby depicting drug use better.

Comorbidity was determined using a modified version of the Functional Comorbidity Index (FCI) [23]. The geriatricians identified diagnoses (rheumatoid arthritis and other inflammatory connective tissue diseases, osteoporosis, diabetes, chronic asthma or chronic obstructive pulmonary disease (COPD), coronary artery disease, heart failure, myocardial infarction, stroke, depressive disorder, visual impairment, hearing impairment, Parkinson’s disease or multiple sclerosis and obesity) based on primary care medical records and the investigator determined FCI index, where a higher FCI sum score indicates greater comorbidity.

Frailty status was assessed using the aCGA [13], which is based on the full CGA and developed to detect frailty in vulnerable population. The aCGA can detect problems that may otherwise go undetected, such as difficulty in bathing, dressing and shopping, reduced cognition or depression symptoms [13]. These problems are common among home care clients, although poorly detected. The validated aCGA consists of 15 questions including 3 questions on ADL; bathing, toilet use and transport, 4 questions on IADL; shopping, food preparation, housekeeping and laundry, 4 questions on the MMSE; calculation, reading, writing and image copying and 4 questions on the GDS-15; “Do you feel that your life is empty?”, “Do you feel happy most of the time?”, “Do you often feel helpless?” and “Do you feel pretty worthless the way you are now?”) (Table 1) [13].

**Table 1:** Prevalence of functional, cognitive and depression domains of aCGA and indication for frailty with aCGA

DOMAINS	Cut-off maximum	Prevalence *n* (%)
Functional status (*n* = 257)	≥**1**	**187 (72.7)**
Bathing (ADL)		115 (44.7)
Transfer (ADL)		31 (12.1)
Continence (ADL)		119 (46.3)
Shopping (IADL)		156 (60.7)
Preparing meals (IADL)		170 (66.1)
Housework (IADL)		94 (36.6)
Laundry (IADL)		132 (51.4)
Cognitive status (*n* = 257)	≤**6**	**145 (56.4)**
Attention and Calculation (MMSE)		38 (14.8)
Reading (MMSE)		53 (20.6)
Writing (MMSE		82 (31.9)
Copying (MMSE)		116 (45.1)
Depression (n = 257)	≥**2**	**146 (56.8)**
Emptiness (GDS)		65 (25.3)
Happiness (GDS)		26 (10.1)
Helpless (GDS		117 (45.5)
Worthless (GDS)		81 (31.5)
INDICATION FOR FRAILTY (*n* = 257)	Positive score on ≥1 domain	231 (89.9)
	Positive score on ≥2 domain	162 (63.0)

Boldfaced text and number are not significance

### Statistical analysis

The prevalence of frailty was assessed in all three aCGA domains: functional status, cognitive status and depression. The cut-off value for functional status was ≥1, for cognitive status, ≤ 6 and for depression, ≥ 2. All three domains were unified into one frailty variable by which the participants were categorised into two groups. The frailty variable indicates frailty with a positive score in the ≥1 domain. The frailty variable was also calculated with a positive score in the ≥2 domain [14]. Statistical comparisons between the groups were done using a chi-square or t-test and a Mann-Whitney U-test for non-parametric variables, with a *p*-value of 0.05 considered significant. The results were expressed as frequencies with percentages or means with standard deviations (SD). Univariate and multivariate regression analyses were performed to identify demographical, clinical and functional factors associated with frailty. The analyses were done using SPSS version 21.0 (SPSS, Inc., Chicago, IL).

## Results

The mean age of the home care clients was 84.5 (SD 5.3) years and 73.9% of them were female. Of the 257 home care clients, 231 (89.9%) were classified as frail using the aCGA in the ≥1 domain and 162 (63.0%) in the ≥2 domain (Table 1).

The home care clients who were classified as frail according to at least 1 point in the ≥1 domain had statistically significant fewer years of education, poorer self-rated health, higher comorbidity and greater hearing impairment and they were more frequently at risk of malnutrition or malnourished than non-frail home care clients and unable to walk 400 m independently (Table 2). The results were similar when using at least one point in both the ≥1 and ≥2 domains, except for hearing problems. In this domain, the home care clients more often had a diagnosis of stroke and visual impairment. Mean BMIs was a little higher in the frailty group compared with the no frailty group.

**Table 2:** Participants’ characteristics and functioning by frailty

	Positive score on ≥1 domain			Positive score on ≥2 domain		
	Frailty *n* = 231 (89.9%)	No frailty *n* = 26 (10.1%)	*p* value	Frailty *n* = 162 (63.0%)	No frailty *n* = 95 (37.0%)	*p* value
Demographic characteristics						
Female	167 (72.3)	23 (88.5)	0.075	117 (72.2)	73 (76.8)	0.415
Age, mean (SD)	84.7 (5.28)	83.3 (5.45)	0.220	85.0 (5.37)	83.8 (5.14)	0.105
Living alone, *n* (%)	153 (66.2)	18 (69.2)	0.759	108 (66.7)	63 (66.3)	0.954
Education, mean (SD)	7.84 (3.11)	11.20 (4.79)	<0.001	7.41 (2.83)	9.47 (4.0)	<0.001
Clinical characteristics						
Poor self-rated health, *n* (%)	66 (28.8)	2 (7.7)	0.021	56 (34.8)	12 (12.8)	<0.001
Risk of malnutrition/malnutrition, *n* (%)	194 (88.2)	16 (61.5)	<0.001	140 (92.1)	70 (74.5)	<0.001
Drugs in regular use, ≥ 10, *n* (%)	126 (57.0)	13 (52.0)	0.632	94 (61.4)	45 (48.4)	0.045
FCI, mean (SD)	3.01 (1.87)	2.12 (1.75)	0.023	3.16 (1.84)	2.53 (1.86)	0.006
Heart disease, *n* (%)	139 (62.9)	15 (57.7)	0.604	99 (64.7)	55 (58.5)	0.329
Diabetes, *n* (%)	67 (30.3)	8 (30.8)	0.962	45 (29.4)	30 (31.9)	0.678
Asthma / COPD, *n* (%)	50 (22.6)	4 (15.4)	0.398	39 (25.5)	15 (16.0)	0.078
Stroke, *n* (%)	60 (27.1)	3 (11.5)	0.084	49 (32.0)	14 (14.9)	0.003
Visual impairment, *n* (%)	58 (26.2)	7 (26.9)	0.941	47 (30.7)	18 (19.1)	0.045
Hearing impairment, *n* (%)	42 (17.0)	0 (0.0)	0.015	28 (18.3)	14 (14.9)	0.489
BMI, mean (SD)	27.4 (5.59)	25.2 (3.9)	0.063	27.5 (5.55)	26.6 (5.30)	0.206
Functioning						
Walks 400 m independently, n (%)	126 (55.0)	26 (100)	<0.001	73 (45.3)	79 (84.0)	<0.001

*SD* Standard deviation, *BMI* Body Mass Index, *MNA* Mini Nutritional Assessment, *FCI* Functional Comorbidity Index, *COPD* Chronic obstructive pulmonary disease

The results from logistic regression models (Table 3) showed that, in multivariate analysis, the risks of malnutrition or malnutrition OR = 4.27, 95% CI = 1.56, 11.68) and low level of education (OR = 1.14, 95% CI = 1.07, 1.23) were independently associated with frailty in the ≥1 domain. Similar results were found in the ≥2 domain (OR = 3.36, 95% CI = 1.62, 8.40 and OR = 1.13, 95% CI = 1.07, 1.20).

**Table 3:** Univariate and multivariate association between patient characteristics and frailty

Variable	Positive score ≥ 1 domain	Multivariate OR (95% CI)	Positive score ≥ 2 domain	Multivariate OR (95% CI)
	Univariate OR (95% CI)		Univariate OR (95% CI)	
Sex (female)	0.34 (0.10–1.17)		0.78 (0.44–1.41)	
Age	1.05 (0.97–1.14)		1.04 (0.99–1.09)	
Living alone	0.87 (0.36–2.09)		1.02 (0.59–1.74)	
Education	0.81 (0.74–0.90)^b^	0.83 (0.75–0.92)^b^	0.84 ((0.77–0.91)^b^	0.83 (0.76–0.91)^b^
Poor self-rated health	4.86 (1.12–21.14)^b^		3.64 (1.83–7.25)^b^	
Risk of malnutrition/malnutrition^c^	4.66 (1.92–11.35)^b^	4.27 (1.56–11.68) ^b^	4.00 (1.89–8.47)^b^	3.69 ((1.62–0.40)^b^
Drugs in regular use ≥10	1.22 (0.54–2.80)		1.70 (1.01–2.86)^b^	
FCI	1.35 (1.04–1.75)^b^		1.21 (1.04–1.40)^b^	
Heart disease	1.24 (0.55–2.84)		1.30 (0.77–2.20)	
Diabetes	0.98 (0.41–2.36)		0.89 (0.51–1.55)	
Asthma / COPD	1.61 (0.53–4.89)		1.80 (0.93–3.49)	
Stroke	2.86 (0.83–9.86)		2.69 (1.39–5.22)^b^	
Visual impairment	0.97 (0.39–2.42)		1.87 (1.01–3.47)^b^	
BMI	1.09 (1.00–1.18)		1.03 (0.98–1.08)	

^a^Forward Wald selection. Only variables that entered the model are shown

^b^Statistically significant (*p* < 0.05)

^c^The nutritional status was performed using the Mini Nutritional Assessment (MNA

OR = odds ratio; FCI = Functional Comorbidity Index; COPD = Chronic Obstructive Pulmonary Disease; BMI = body mass index

## Discussion

We found that frailty was common among home care clients. A risk of malnutrition or malnutrition and a low level of education were associated with frailty. In previous studies among community-dwelling older people the prevalence of frailty has varied between 4 and 59% [24]. These differences in prevalence may result from different tools used to measure frailty.

The aCGA was developed to pre-screen older patients to determine who would need the entire Comprehensive Geriatric Assessment (CGA) [13]. The aCGA is time-saving and inexpensive compared with the entire CGA [25]. As the aCGA is sensitive and seems to categorise pre-frail cases into the frailty category, this might explain the relatively high prevalence in this study [13]. Also, the fact that home care clients are evidently in more need of help than other community-dwelling older people might explain the higher prevalence in this study compared with studies among community-dwelling older people. The aCGA has not been validated in home care clients. However, it has been used to assess, for example, the frailty status of patients with cancer [13, 14] and without cancer [13, 14], so the aCGA is suitable for this vulnerable population.

Another explanation for the higher prevalence of frailty might be differences in the ages and demographics of the study populations [23, 26, 27]. In several frailty studies the inclusion criteria of the participants’ age has been 65 years, which is ten years less than in our study [26]. As in this study the mean age of the participants was 85 years, the high prevalence can be partly explained by the fact that the prevalence of frailty increases with age [2, 24, 28].

The risk of malnutrition or malnutrition was associated with frailty. A German study among community-dwelling older people aged 75 or older also found this association [29]. Abellan Van Kan and Vellas suggested that MNA scores of 17–23.5 could identify frail old people and that the MNA could be used to screen for frailty [30]. Furthermore, unintentional weight loss is one symptom of frailty [3], and rapid weight loss is a severe problem among community-dwelling older persons who are at risk of malnutrition or malnourished [31, 32]. So, the phenomena of frailty and sarcopenia often overlap [5]. Muscle mass is low in sarcopenia and poor nutrition further accelerates loss of muscle mass. This results in decreasing physical functioning, causing balance and mobility problems, falls and weak muscle strength [12, 32, 33]. For home care clients, the importance of nutrition and muscle strength should be emphasized.

In this study, frail persons have slightly higher BMI values. Although their BMI is higher, it is nevertheless in line with the recommendations. According to an epidemiological study [34], the recommended BMI for a person over 70 years of age is 24–29. Winter et al. [35] found in a 32-study meta-analysis a greater risk of mortality risk in those with a BMI < 23.0, which is partly in the WHO overweight range (BMI: 25.0–29.9). On the other hand, overweight and obese older persons can also be frail [36, 37].

There also was an association between education and frailty. Older persons with less years of education had a higher prevalence of frailty than persons with more years of education [37, 38]. This should be considered a public health issue and should be taken into account when focusing preventive intervention in clinical practice.

The strengths of this study were the population-based sample of home care clients and the multi-scientific approach. The study population is comparable to home care clients in Finland. A well-trained team of specialists collected the data. Additionally, the present study had no exclusion criteria. A limitation of this study could be its cross-sectional design; the risk factors and frailty may not have a cause-effect relationship.

## Conclusions

Frailty is a common health problem among home care clients. A risk of malnutrition or malnutrition and a low level of education were associated with frailty. Screening for frailty is needed to detect the most vulnerable old people to prevent further adverse health events.

## Abbreviations

aCGA: Abbreviated Comprehensive Geriatric Assessment
ADL: Activities of Daily Living
BMI: Body mass index
CGA: Comprehensive Geriatric Assessment
CI: Confidence interval
COPD: Chronic obstructive pulmonary disease
FCI: Functional Comorbidity Index
GDS-15: 15-item Geriatric Depression Scale
IADL: Instrumental Activities of Daily Living
MMSE: Mini-Mental State Examination
MNA: Mini Nutritional Assessment
NutOrMed: Nutrition, Oral Health and Medication study
OR: Odds ratio
SD: Standard deviation
